# Supplementary material for: A realist perspective on optimizing community health workers’ roles and functions to deliver integrated people-centred care
Source: PLOS Glob Public Health. 2025 Sep 3;5(9):e0004926. doi: 10.1371/journal.pgph.0004926 (PMC12407478; doi:10.1371/journal.pgph.0004926)
Supplement: S1 File — (DOCX) [file pgph.0004926.s001.docx]

**In-depth Case Descriptions of Five Communities in KwaZulu-Natal, South Africa**

| **Abbreviation** | **Name** |
| --- | --- |
| TYS | Taylors Clinic |
| GME | Gomane Clinic |
| GUA | Gcumisa Clinic |
| BUE | Bruntville Community Health Centre |
| CAD | Crammond Clinic |

**Bruntville Cases**

***Case 1 – Bruntville***

The peri-urban township of Bruntville shows characteristics of high unemployment rates along with socio-economic difficulties and restricted access to essential services. The township features a combination of formal and informal residential areas and essential facilities which include a central clinic together with local schools and a community hall serving as War Room meeting space and a police station which remains out of reach. Community Health Workers (CHWs) operate under these conditions, providing basic health support, conducting household visits, and coordinating referrals with overstretched and distant government services such as the Department of Social Development (DSD).

The CHW daily tasks involve meeting patients, establishing trust and delivering both clinical services to check children’s development and administer vitamins and deworming while providing social support for medication adherence, helping people obtain identity documents, and social grants. The CHWs faces geographical challenges and safety threats in this community, and administrative delays when connecting communities with DSD or local clinics.

The interviewed household consists of multiple generations under the leadership of a disabled woman who receive social grant and who is raising her children and grandchildren who require ongoing medical treatment. The family members demonstrate excellent internal bonding through their daily practices of mutual support to help each other with medication adherence. The family head explained how household members frequently check on each other to remember their medication schedules and uphold their healthcare plans. However, the household members maintain their supportive bond only within their immediate family boundaries. Meaning, the family handles all their problems independently instead of reaching out to external sources for help, because they prefer handling matters within their family unit. Despite this family bond, the family faces a challenge with one family member. A young woman who avoids taking her daily prescribed medication despite regular encouragement from others because she chooses to focus on social media activities. This situation creates an intense emotional stress for the caregiver of this household, who then accentuates the need for CHWs and NGOs to provide external support.

In this community, people living with chronic illnesses do not receive any assistance from NGOs or community-based organizations and the household members reported a complete absence of such support. The household members requested external support for follow-up activities and encouragement because they need help to maintain regular medication use. The CHW who operates in the same environment describes facing both high demand and emotional challenges and logistics difficulties in her work. Many homes welcome and respect her but she continues to deal with non-cooperative clients who hide their diagnoses because they fear stigma or job loss. During her home visits she experiences uncomfortable and emotionally challenging situations and performs functions that exceed her formal role or responsibilities by connecting communities with DSD and document retrieval from the Home Affairs Department, activities which CHWs are not trained to perform.

The treatment adherence becomes more complex because of side effects from medications and food scarcity. Patients experience dizziness together with other medication side effects and food shortages make them avoid taking their medication on a regular basis. The CHW actively continues her work despite these challenges by making treatment collection arrangements and motivating patients to avoid defaulting from their treatment. Households must travel extended distances to reach clinics and administrative offices and the essential police service remains both physically and socially remote from the community.

The area lacks any NGO or CBO programs for health education delivery and home-based care reinforcement and adherence support. War Room involvement exists but its consistency and inclusion levels vary. CHWs function as de facto case managers by handling all patient needs, spanning from clinic coordination to social services. Ultimately, this particular case from Bruntville demonstrates how CHWs play an essential role in maintaining healthcare continuity while they function with scarce resources coupled with undefined professional boundaries and intense emotional strain.

***Case 2 Bruntville***

The township of Bruntville located in KwaZulu-Natal functions as a semi-urban settlement where residents maintain close bonds with each other but struggle with major social and health problems. Residents of this area need to visit the town for both educational facilities and police services because these services are unavailable inside the community. The social workers and electricity purchasing points operate from town which is reachable through a R10 transportation cost yet this limitation affects people who have movement difficulties or ongoing medical conditions. The local residents purchase their food at spaza shops and CHWs from the community encourage them to cultivate vegetable gardens to enhance food security and improve dietary habits that benefit chronic conditions control.

The CHW at Townview in Bruntville resides in the same neighborhood where she delivers services which allows for better accessibility and community bonding. The CHW makes home visits to detect health and social issues through targeted support of patients with chronic conditions such as HIV and diabetes. She provides households with information about regular clinic visits as well as treatment adherence and healthy lifestyle practices. The CHW uses her health promotion training to educate people about diet modification which involves salt and fat restriction as well as spice limitation to support patients with chronic diseases through condition-specific dietary guidelines. Furthermore, the CHW creates referral letters when clinic visits become necessary.

The CHW in this case travel to her service area by foot through familiar paths which include Fanzo’s and Luh’s tuckshops as recognizable community points. The closeness of geolocation of residential areas allows the CHW to execute her work more efficiently because she can respond swiftly to emergencies. In addition, her deep connection with the community enables her to keep in touch with clients who occasionally seek help from her residence – further reinforcing accessibility. The CHW in this area also verifies whether the client receives support from family members and makes sure both caregivers and patients understand their condition management requirements.

The health and social problems in Bruntville are quite severe. The most prevalent medical condition in the area is HIV followed by hypertension and diabetes being less common. The clinic faces frequent cases of medication defaulting mainly among youth with HIV who avoid clinics because they fear disciplinary measures and stigma. Drug and alcohol substance abuse constitutes a major problem in the community. The area experiences house burglaries that young men who use substances commit. Safety concerns during home visits lead CHWs to work in pairs specifically when visiting households that contain young male residents.

Non-governmental organizations deliver medication support yet the community lacks comprehensive integrated assistance. The CHWs have not receive any visible NGO support for community-based programmes. Social workers maintain their office in the town and fail to become fully integrated within the daily community environment.

The community members express strong appreciation for their CHWs despite facing multiple difficulties. For example, a woman who suffers from diabetes and experiences regular swelling in her legs considers her community health worker to be essential. The CHW provides medication delivery services and performs regular monitoring of her medical state. Also, the close living arrangement between them enables the CHW to provide initial support before the woman travels to the clinic by offering to collect her medication for her at the clinic. However, tensions exist within the system. Clinic staff members regularly deny CHWs' medication requests for patients by demanding the patients' personal attendance. This erodes the effectiveness of CHWs and contradicts their purpose to enhance accessibility.

The CHW's success in reaching homes is enhanced because she works in the same location where she resides. Her knowledge of the households combined with her established relationships with clients helps build trust which results in better care responses. Clients can contact her whenever they require assistance while she provides immediate referrals to the clinic or writes referral letters for urgent situations. The close physical location combined with her knowledge of the community allows patients to avoid waiting for care or visiting the clinic directly after seeking her assistance. The case illustrates the significant but restricted position of CHWs in underserved populations.

Ultimately, the CHWs deliver highly individualized care through their community-based practice but face challenges from safety hazards and service gaps and variable institutional backing. Their position within communities allows them to connect health systems with everyday life in a way that few other interventions can achieve.

***Case 3 – Bruntville***

The area of Phumlas in Bruntville exist as a semi-urban settlement within KwaZulu-Natal which exhibit high topography alongside limited infrastructure and economic challenges. The locations contain a large number of elderly people together with orphaned children and jobless youths. The distance from health and social services together with transportation expenses create challenges for access to essential services. The health and well-being of residents depends heavily on community health workers who function as essential service connectors in these specific settings.

At the beginning of their workweek CHWs record their attendance at the clinic followed by daily activities that include home visits and assistance with health referrals and system navigation for Home Affairs ID and birth certificate applications and SASSA social grants. Community health workers provide support across medical domains along with general social assistance through their services for residents who lack access to electricity and water services and for people who have lost their homes.

The local community members recognize CHWs without exception except for interactions with new community members. During household visits they introduce themselves as personnel who help with any matter concerning the community. When they observe a child without a birth certificate they will determine the delivery location to check if proper documentation exists. Hospital births often lead mothers to forget child registration after discharge especially during traumatic or overwhelming birth events. The CHWs maintain oversight of paperwork documentation and lead families to the nearest registration facilities thus preventing extensive journeys to places such as Escourt or Maritzburg (approximately 100km away). Additionally, the CHWs help elderly pension recipients who face unexplained payment stops by creating reference documents which direct them to visit SASSA offices for correction.

Chronic illness management depends heavily on CHWs as they play an essential part in this process. The CHWs regularly meet patients who have HIV as well as diabetes and hypertension and other medical conditions to help them maintain their complex medication schedules. Patients frequently battle to handle their multiple medical conditions while also losing track of their medication and trying different schedules because of pill exhaustion and misunderstanding. Some patients abandon their medical treatments while also halting selected prescriptions or misinterpret their medication timing. Patients experience confusion because the pill colors and packaging designs periodically change and this effect is most pronounced among elderly patients. The confusion regarding medications spreads to CHWs because they lack knowledge about new medications and they want periodic training sessions.

Training programs for CHWs remain outdated and they only receive basic HIV treatment and general home-based care instructions from years ago. CHWs strongly wish to receive continuous training specifically for medication management and new disease protocols. CHWs maintain a broad range of responsibilities yet they do not receive official recognition as healthcare workers. The staff members lack official employment status which denies them access to pension payments and UIF benefits and creates a fear that they will become unsupported when they retire or pass away. Through their 15 to 20 years of work they express disappointment that their dedication will end without any official recognition or financial stability.

The awareness of CHW presence among residents becomes uncertain especially when senior officials are not present. Households show mistrust toward CHWs until they see a nurse visiting or when the worker arrives in an official vehicle. These residents express their job-related dissatisfaction by hoping their unemployed children could secure employment through denying recognition of existing CHWs (wanting their own unemployed children to become CHWs hence denying CHWs access so that they may lose their jobs). People in the community withhold their support through various means which indicate problems related to politics as well as desperation and poor communication.

The elderly woman in Phumlas maintains a home with multiple orphaned grandchildren who are descendants of her deceased sons. The grandmother has survived all her relatives and relies on both her grandchildren and neighbours for care. The daughter who works in another town sends her own children to help. She receives financial support from her pension along with sporadic assistance from her son who resides in Pretoria and constructed her home. The house remains simple in this area because previous housing opportunities failed to materialize while numerous structures exist in a state of decay or self-made construction. The patient deals with multiple health issues because she used to walk to the clinic with her walking stick but she now needs a wheelchair while receiving assistance on her visits. Community health workers provide the medication to her. Medical restrictions make it difficult for her to follow her diet plan because Future Life porridge costs too much so she uses maize meal instead which creates negative effects on her health.The neighbors play an essential role in providing her with support in her daily life. The community members regularly check on her while they also prepare her meals and perform household duties when her family is absent. The elderly woman remembers how she used to help her neighbors when she was younger and now recognizes their care as the return of that previous kindness.

The safety conditions in these communities stand as a major priority for everyone. The youth who engage in drug activities are known as "paras" and create safety risks. Young women experience fear about traveling alone for work because of violent incidents and theft that occur in their community. The existing infrastructure and roads remain in poor condition while development promises remain unfulfilled. The community does not have any nearby schools or formal care centers or stable government support. The elderly woman explains that CHWs serve as the only dependable support while social workers remain absent from the community. The CHWs continue to serve as the primary support system but their unsafe working environment and insufficient backing reveal how vulnerable care services are in these areas.

**Cramond Cases**

***Case 1 - Cramond***

This case presents a situation in which a family in a rural setting encounters health services challenges with both geographic and social-economic constraints affect their availability. The family lives in a difficult environment with dysfunctional roads along with limited healthcare services. The clinic serves as an important healthcare resource although it remains difficult to reach because of current infrastructure conditions. The household contains six people who include both a pensioner who leads the family as head and various members suffering from chronic conditions including hypertension and diabetes. Socioeconomic challenges from crime rates and service limitations affect the community while the members demonstrate some strength through minimal local assistance. The challenges notwithstanding, there exists a certain level of community resilience which is enabled by scarce local resources and available support networks.

A pensioner who leads the family supervises the medical needs of all family members with chronic illnesses. The pension money serves as the primary income source for the family but it does not provide enough funds to satisfy all their requirements. The family depends on their neighbors to transport them to medical facilities and to occasionally deliver elderly care supplies including diapers. CHWs function as essential providers of healthcare support within the family unit. The CHWs conduct scheduled medical examinations while teaching health-related information and providing healthcare access when family members cannot access the clinic because of distance or financial difficulties. The CHWs efficiently handle short-term health concerns although they face resource limitations which restrict their capacity for long-term solutions.

The family encounters numerous obstacles when trying to obtain sufficient medical care. The clinic serves as the main healthcare facility but its location far from the residence combined with deteriorated roads creates transportation difficulties. The elderly members of the household need help to get to the clinic since they depend on rented vehicles from neighbors which creates additional expenses. The family faces difficulties managing their chronic diseases because they struggle with both medication expenses and wheelchair maintenance which became impossible because of the poor road conditions. The family faces problems when dealing with illnesses at home because they lack sufficient healthcare professional availability. The CHWs assist in filling this gap but they cannot always respond to urgent situations.

The CHW plays an essential function in this particular context. These workers offer vital health education together with disease prevention guidance and connections to advanced medical care. The CHWs experience multiple difficulties because they receive insufficient training and have restricted access to healthcare services while managing various healthcare needs across a large population with minimal support. The mobile clinic service runs infrequently while failing to access remote locations which creates an outreach service deficiency.

The community deals with serious social issues and economic problems apart from their health-related difficulties. High crime rates in the area create an environment of insecurity that makes life uncomfortable for community members. The residents need to exercise caution during their movements since theft remains a persistent danger. The safety concerns create additional psychological distress for people with chronic diseases because they must protect themselves from both health-related issues and violence. These families experience additional difficulties because of the unstable infrastructure which prevents them from getting reliable access to water and electricity. The families endure water shortages that continue for weeks while power outages create disruptions to their daily routines including their healthcare and cooking needs.

Local support for day-to-day needs exists through tuck shops and informal networks even though large-scale institutional help remains absent. Local government services operate with minimal capacity in their responses toward addressing community needs. Local residents must wait for extended periods while authorities work on housing improvements as well as healthcare service delivery. The community members actively work to push for enhanced service delivery despite their present difficulties. Community members submit reports about issues to local authorities yet receive inadequate and delayed responses. Various families seek disaster relief along with home reconstruction assistance because of natural disasters but they doubt these programmess will deliver satisfactory results.

The rural community continues to experience difficulties in securing improved healthcare and social services due to an ongoing large gap between available services and required services. Multiple factors related to healthcare access and socio-economic conditions and community support systems create a complex situation which needs diverse solutions. The CHWs lead the way but require expanded resources and backing to properly serve their community needs.

***Case 2 - Cramond***

Residents in Thokozani Township Section D Crammond of KwaZulu-Natal experience their daily life through the combination of enduring difficulties and neighborhood-based support networks. The semi-rural location features houses spread across various streams and steep terrain and railway lines which make it hard to reach nearby facilities such as the clinic and water office and community halls. Within this community setting, CHWs play an essential role by connecting formal healthcare services with residents who need illness care and support and those with limited access.

CHWs perform their duties through walking while they check on homes to teach residents about hygiene and HIV and tuberculosis (TB) prevention and direct them to visit clinics when needed. The essential contributions of their work face regular undervaluation from others. Better financially stable residents together with others choose to ignore the CHWs because they believe these workers bring only disturbances instead of help. CHWs actively seek out homes with noticeable neglect and ongoing illnesses to provide specific health education and emotional support despite facing challenges. Several problems within the structure add to the existing difficulties. Identity documentation problems are widespread, with many children unable to attend school due to a lack of birth certificates. Children lack official recognition along with basic service access because their parents failed to register their births and because forged documents remain unresolved. The CHWs dedicate their efforts to both health support and administrative advocacy because the community encounters frequent failures from bureaucratic systems.

Family members play an essential part in delivering care to their households. For example, in this case a grandmother takes care of her mentally ill grandson by giving him medicine and food and using a measured communication style which she learned at the hospital. The grandmother's caregiving practice combines medical advice from hospital visits with ongoing CHW interactions. Family systems frequently experience intergenerational conflicts as youthful members who struggle with substance abuse or chronic diseases display poor compliance with their medical treatments and show resistance to outreach programs. The community members who are older adults show better cooperation and show regular commitment toward managing their health. The involvement of youth in support programs faces regular breakdowns. Health group establishment and educational events usually do not gain interest unless participants expect tangible advantages. Youth members hold doubts about these initiatives because they believe these programs fail to deliver substantial benefits. The community members who are older demonstrate better openness to participate in activities that support their health since they serve as health advocates for their families to encourage hygiene practices and medication use and clinic attendance.

CHWs deliver essential services including medication monitoring and emotional counseling and assistance with clinic access despite the barriers they face. The workers engage in multiple responsibilities that extend past healthcare by establishing trust and resolving family disputes while improving household communication. CHWs organize patient care by bringing family members into the process or providing their own assistance to maintain daily medication schedules for patients who have multiple health issues or bedridden conditions. The CHW receives preference for assistance from patients who have strained relationships with their family members because they view the CHW as an unbiased person who shows proper respect. CHWs maintain unorganized communication channels with their superiors. The CHWs lack internal referral forms so they must call their supervisors directly through phone calls or private messages for assistance with difficult cases. The absence of proper organizational structure mirrors bigger institutional problems which force CHWs to operate without official tools while they handle complicated situations without proper administrative support.

The local community uses informal institutions as their main foundation for support systems. Local residents show strong appreciation for both CHWs and police forums and church organizations which deliver emotional backing and practical assistance. An elderly woman who used to attend a senior citizens club shares how the club offered physical exercise and practical education and emotional support especially to abused and stressed grandmothers. Her arthritis and restricted movement prevent her from taking part anymore yet the essential role of these spaces in maintaining mental and physical wellness remains evident. The accessibility problem persists as a crucial matter. Patients need to traverse rivers and ascend hills while dealing with dangerous paths to reach the clinic. A few patients depend on CHWs for both transportation arrangement services and medication pickup services.

The clinic workers along with CHWs track down residents who stop taking their medication through home visits and personal meetings when residents become disengaged from treatment due to alcohol misuse or loss of faith. The follow-up procedures stem from personal connections between healthcare providers and patients together with mutual respect and their collective dedication to wellness. The health and social environment of Thokozani exists because of its interconnected social bonds. The health of the community endures because CHWs and dedicated community members work together through a cooperative spirit while the formal healthcare facilities continue to be inadequate. The efforts of these workers demonstrate how the system depends on informal care that combines improvisational approaches with traditional local knowledge and community-based resilience.

***Case 3 – Cramond* – *(There is no OTL but CHWs are supervised by the OM)***

The community in Cramond KwaZulu-Natal faces poor living conditions because residents lack employment opportunities and struggle to obtain basic services. The area presents itself through substandard homes that include both unofficial structures and neglected permanent residences as residents primarily survive on social benefits together with casual trading activities and minimal public services. The household features a retired pensioner who resides with his younger wife who stays home while attempting to sell goods at school to make ends meet. The elderly couple provides caregiving duties to their grandkids after their daughter passed away. The building suffers from structural damage which causes water to enter through the roof. The elderly man has been waiting for years to receive housing assistance through the registration process yet he has not received any response from officials who continue to ignore his follow-ups which intensify his disappointment and feeling of being abandoned. The man travels to the nearby clinic to receive medical care but he faces inconsistent access to broader health and social services despite the close location. The man handles his multiple chronic diseases effectively by managing his prescribed medications but he experiences irregular support from community health workers (CHWs).

The CHWs who are supposed to serve his area show very little presence since he has not seen them in the past eight months. The CHWs conduct superficial visits which do not include practical support. He understands the CHW's past efforts which included arranging mobile clinic services and food parcel distribution at a community hall for residents. The interventions he receives are unusual because they do not occur as standard procedures. The CHW reports that her daily responsibilities involve multiple tasks including four household visits to check medication use and child development as well as home condition assessment and social vulnerability identification through tests for food security and identity documents. The CHW handles various responsibilities that range from inspecting vitamin A supplements in children to advising teenagers about pregnancy testing and reminding adults with chronic illnesses to follow their treatment plans. She maintains connections with social workers and schools and the war room—a local intersectoral government forum—but the effectiveness of these collaborations is limited by poor departmental attendance and follow-through.

The area faces a growing youth unemployment crisis because many young people still work in limited EPWP municipal positions and farm jobs are disappearing due to declining agricultural operations. The surrounding white-owned farms used to employ many workers but agricultural operations decreased leading to a disappearance of these employment opportunities. Young people spend their days aimlessly while some earn the derogatory label of "paras" which suggests substance abuse or criminal behavior thus worsening their sense of desperation and vulnerability. Crime levels in this section are not overwhelming to residents who report feeling secure in their community. People have accepted their fate to live independently since the expression "every man for himself" becomes a recurring theme.

Government clinics exist along with a police station and food parcel distributions from DSD and NGOs Thembalihle and Sisonke but services remain unreliable and sporadic and often insufficient. The CHW is disappointed because war rooms which used to function as support centers now lack complete departmental attendance which makes it hard to obtain assistance for families without financial resources or identification documents. The residents face challenges in obtaining seeds and agricultural support to cultivate food even though they received encouragement to develop home gardens. The existing climate forces CHWs to deliver complete household-level support although they need constant institutional backing to effect meaningful changes in lives.

The overall dynamic is one of survival in a resource-constrained setting where local actors—especially CHWs—play a crucial but undervalued role. The elderly man and other community members understand the concept of support but they feel disregarded by the programs created to help them. People survive in this environment by relying on family support networks and occasional food assistance from a tuckshop yet they exist mostly in need rather than dependable service.

**Gcumisa Cases**

***Case 1 – Gcumisa***

A woman who recently moved to Mahlathini, Swayimani from Nkululeko because of her health problems and personal safety issues now stays with her daughter-in-law and grandchildren. The student who studies far from home realized that staying alone was dangerous for her because she cannot even cook or clean or bathe herself. Her present setting is better because it has paved roads and family members nearby but she is still unable to take care of herself. Her mobility is limited and she does not have the R30 for a round trip taxi fare to the clinic. She has not been able to make it to her health care appointments on a regular basis. She has several chronic diseases including diabetes and hypertension and her health is deteriorating rapidly. The daughter-in-law takes care of her during the day but she also has to attend to the house and the garden. It is difficult for the daughter to do it and the family is not able to give her the meals she needs and the personal care she requires. The elderly woman showed a great deal of fear and anxiety about not being able to take care of herself and the lack of help on the days when the caregiver is not available. She wishes for someone to assist her with bathing, cleaning and cooking because she knows that her needs are more than her current living situation can meet. The problem of access to health care in the area is because of distance, no transport, and high costs compared to the income.

There is no local organization or any formal home-based care services except for the community health workers. The CHWs work in difficult conditions as they traverse unsafe routes which are often used by loiterers who are a threat to the safety of women on their own. Despite the risks they encounter they go to several households every day to teach about hygiene, diet, and disease management. They conduct an evaluation of each home separately and modify their teaching methods according to what they can see, and in some cases they may assist with tasks that are physically demanding like bathing patients who are bedridden, cleaning houses or preparing meals. These CHWs also act as a link between the community and the local coordinating body which is called the war room where they refer the needs that are outside the health sector such as food insecurity and the need for disability grants.

The structures that used to bring medication to community points for easier collection are no longer in place as they were not sustained and people had to go to distant clinics to get their medication. In some cases, the health workers will go to get the medication for the patient if the patient is too sick or weak to go themselves. The work in the community is also influenced by gender relations and social structures. It is not easy to educate men especially in public and often in taverns where they are drunk and may be hostile or laugh at the person trying to educate them. This atmosphere does not allow for effective interaction and CHWs have to pull out and try again at a later time under different circumstances.

In the household, not all patients are open about their condition especially with the stigmatized diseases like HIV. Therefore, health care is mostly reactive and is only sought when there is a visible deterioration and not preventive. However, the presence of CHWs is a source of relief and optimism. The elderly and the needy look forward to their visits as it means that they will be taken care of and that they will receive better care. It is a difficult and exhausting work that is done in the poor settings with no protection at all but it is still important in filling the gap between the formal health care system and the most vulnerable people in the society.

***Case 2 – Gcumisa***

Residents in Swayimane, a rural area of KwaZulu-Natal, live in separate homes and encounter multiple barriers in receiving healthcare. The Gcumisa Clinic operates as the main healthcare center but many homes remain distant from its reach so patients need to walk extensive distances or use unreliable public transportation. The taxis operating in the area tend to disregard elderly or sick individuals which forces them to continue walking despite their discomfort. The community faces severe poverty alongside high unemployment rates which causes most families to survive through old-age pensions and disability grants. The death of grant recipients together with missing ID cards and birth certificates leaves families exposed to vulnerability and without any support. The combination of rising living expenses and insufficient food supply with disease burden creates difficult conditions for daily survival. The economic challenges faced by the community affect health behaviors because individuals skip their prescribed medication because they cannot afford food or lack transportation or must wait extended periods at clinics. The community health workers stand as vital figures in these situations. Through home visits they check for patients' medication adherence and retrieve prescriptions at the correct time. The workers attempt to know the reasons behind defaulting while simultaneously assisting patients to understand barriers such as food scarcity or prolonged clinic waiting times and discriminatory treatment from clinic staff. The healthcare workers promote home gardening through government programs including “One Home, One Garden” to enhance food security through helping people develop small gardens for eating before medicine consumption. These workers dedicate their efforts despite numerous system-level obstacles they encounter. The medical supplies including gloves along with nappies and wound care materials are frequently absent from their resources. The healthcare staff receive instructions to care for patients who are bedridden and perform personal care duties yet they lack the essential equipment for proper service delivery. Their professional tasks include referrals and they maintain a standard form for sending patients to clinics or government departments. The patient information forms contain problem details which health workers must verify by following up on clinic visits and treatment received by patients. The clinic nurse or government official uses the back of the form to document patient encounters by confirming patient visits and recording the implemented interventions. These forms serve as a reference for every type of referral including SASSA as well as physiotherapists while creating a chain of responsibility. The health workers need to locate patients to retrieve feedback forms after their clinic visits since the feedback system is inconsistent. The war room meetings function as a coordination platform where education department representatives and social development team members and police officials work together with community health staff. The absence of regular departmental attendance creates problems with proper case referrals. Social workers and education officers' absence makes it impossible to handle complex family or school-related issues including child neglect and abuse as well as students who fall behind. The failure of departments to work together leads to many unresolved issues. The lack of interdepartmental collaboration forces community health workers to handle responsibilities outside their normal duties by functioning as liaison officers between families and multiple government departments without proper training or authorization. Numerous household members suffer from ongoing diseases such as diabetes together with hypertension. Elderly residents frequently report symptoms of fatigue and hunger prior to receiving their medical diagnosis. The path to medical discovery along with continuous healthcare for these individuals depends on physical barriers and emotional stress combined with transportation difficulties. Family members cultivate vegetable gardens which produce pumpkin leaves and taro and beans and maize for dietary support and medication compliance. These gardens provide independence to residents because official help networks remain inadequate. Community members express thankfulness towards health professionals who make house calls despite the obstacles they face. Medical support from these visits combines with emotional encouragement for the residents. Many residents complain that social services do not reach their communities to the extent they need. According to the residents the community receives only sporadic assistance from social workers or nurses without any reliable ongoing support. The healthcare system functions in isolated parts which leaves vulnerable populations without access to essential services. The committed community health workers provide hope while maintaining continuity yet the broader system faces resource deficiencies which create uneven and insecure healthcare access in Swayimane.

***Case 3 - Gcumisa***

A community-based health system operates in the rural area of Gcumisa/Swayimani in KwaZulu-Natal to connect clinical services with household-level care. The system depends heavily on Community Health Workers (CHWs) and Outreach Team Leaders (OTLs) who operate in resource-constrained and often hard-to-reach settings. The People-Centred healthcare system relies on these workers as its core members to deliver services through home visits for community members. The assigned CHWs oversee between 45 to 60 households while they make three daily visits to each household. The health workers conduct daily operations by welcoming community members while reintroducing themselves at each stop to establish trust and consistency before delivering educational content about sanitation and nutrition and chronic disease management. The program focuses intensively on supporting people in creating their own small home gardens to produce nutritious sustainable food. The healthcare workers monitor patients' drug compliance while helping patients with chronic illnesses monitor their health through basic tests and they involve younger relatives to assist elder relatives with their medication schedules. The health workers face the challenge of maintaining both compassion and practical solutions when patients resist treatment or present complex illness profiles which requires documenting non-compliance and submitting reports to clinic staff. The OTLs perform dual functions of supervision and direct patient services. Before starting their work they review the next day they will identify which CHWs need help while choosing which homes they will personally check. The staff uses profiling to collect data about household members including their age distribution and employment sources and the educational status of children and healthcare needs of all household members. After conducting household visits they direct patients to clinics and social workers and local councillors. The OTLs link patients to receive TB treatment and antenatal care services while teaching them about HIV prevention and teenage pregnancy avoidance and healthy lifestyle education. The social environment presents a challenging situation for these healthcare providers. The staff members need to handle privacy concerns and public discrimination and maintain household relationships. Young women and some patients avoid disclosing their HIV status or pregnancies to family members and certain clients refuse visits because they want privacy and do not trust the system. To manage this, CHWs adopt creative strategies such as asking patients to “walk them out past the dogs” to initiate private conversations. The health workers encounter multiple logistical issues because they have to walk long distances through bushy areas while facing aggressive dogs and they lack basic equipment including BP monitors and glucose testing kits which were promised but not delivered. These health workers discover purpose and contentment in their daily tasks despite the numerous obstacles they encounter. Through their work health workers develop close bonds with community members and witness behavioral transformations and health progress while serving as educational resources and advocacy representatives. Systemic problems including salary deductions without warning and limited resources and institutional support create both low morale and reduced effectiveness. The CHWs rely on peer support through pair-based coordination for safety purposes while using group chats in addition to weekly Friday clinic meetings for reporting and planning. The healthcare work transcends medical needs to support the community structure. Health workers discover various problems such as unobtained birth certificates and school uniform shortages and family disagreements about financial matters and caregiving responsibilities. The identified social issues get referred to social workers or councillors yet the officials sometimes face challenges because of their high workload. The holistic needs of patients are addressed by CHWs and OTLs who recognize that social factors directly affect medical results. The Gcumisa/Swayimani case shows how trust and resilience face challenges from systemic healthcare gaps in community-based healthcare. Health workers serve as critical links between clinics and households to manage chronic diseases and meet new patient needs and promote dignity through equitable healthcare delivery in environments with scarce resources and recognition. The healthcare system demonstrates a profound commitment to patient-centered care through an approach which reaches beyond medical treatment and administrative procedures.

**Gomane Cases**

***Case 1 – Gomane***

A chronically ill woman living in KwaZulu-Natal faces the dual challenges of household leadership alongside her deteriorating health. As the principal decision-maker at home she retains authority in matters of food preparation and household organization. Her illness has resulted in major changes to the way the household operates. She used to perform cleaning and cooking tasks but now she only does light tasks such as mopping which makes her extremely tired. She developed her illness after her grandmother but the rest of her family members have not gotten sick. The woman continues to determine food selection while maintaining food preparation autonomy through the use of her independent kitchen space within the home. The diagnosis led her to implement substantial modifications in her eating habits. Her doctor recommended she reduce white bread consumption while she chose brown bread as an alternative whenever possible. She independently decided to remove organ meat from her diet and cooking oil because she understood their high fat content. She does not use margarine so she eats her bread plain. She usually boils her diet which includes spinach, unpeeled potatoes, carrots and butternut and beetroot. She eats the plain boiled vegetables as her main meals while sitting alone on the veranda with minimal salt seasoning. She maintains her healthy eating choices even though her children and husband prefer more oil-rich foods. The husband joins her in eating plain boiled meals occasionally even though he faces illness and doctors advised him against eating organ meat. The granddaughter appreciates her grandmother's food but the younger members of the household refuse to eat boiled organ meat when she prepares it. The support system in her life has come from her high school-educated granddaughter. The woman depends completely on the community health worker (CHW) for all needs when her granddaughter leaves because she cannot take care of herself. The CHW delivers cooked traditional wild herbs and performs regular support tasks and emotional support for the patient. The CHW provides complete care by making sure the woman eats her medication and remains emotionally and physically stable. The CHW demonstrates a strong professional connection through her frequent interactions with this household. She starts her daily work by cleaning her home and tending to her garden before starting her house visits at 9 a.m. The number of houses she visits in a day ranges between three to four based on the complexity of cases she encounters. Her tasks involve checking home cleanliness as well as providing support to bedridden patients and monitoring pregnant women while distributing MediPost medications and offering spiritual support. She makes observational notes about household conditions without criticism through diplomatic conversations about cleanliness issues. During her initial visit to sick patient households she introduces herself professionally then collects family member details and asks to meet the ill person. She provides family members with necessary education about patient care which includes bed maintenance and patient handling techniques that protect their rest. The CHW acts as an essential link which connects household needs to health services at the clinic. She assists MediPost patients to register for the program which enables their medication delivery at local centers or right to their home when they need mobility assistance. The clinic congestion decreases through this system and patient care stays continuous. She maintains professional secrecy by avoiding any disclosure of personal matters including family disputes and critical medical situations. The CHW functions as both confidante and advocate through patient-focused emotional intelligence to establish trust and protect household members from feeling exposed or disrespected. A chronically ill woman in the same community explains how she faces challenges in reaching the clinic. She explains the path she takes by pointing out recognizable points such as water tanks and shops and a creche. The distance between her location and the clinic does not cause her distress because she walks with a CHW or receives assistance from outreach team leaders who use vehicles. The clinic staff shows her respect throughout the process while they take her clinic cards to provide immediate assistance. She deeply appreciates the CHW because before this assistance she missed treatments and struggled to obtain medical care because of her physical constraints. The CHW provides her with home-based medical care services along with medication management and continuous health monitoring without requiring her to leave her yard. According to her reports the CHW together with her grandchild represent her only dependable sources of help because NGOs along with other organizations fail to offer support. The household considers the CHW as their most important resource because she delivers both medical support and maintains their dignity while making them feel included. This case demonstrates the complex connection between long-term illness and rural household situations while showing how limited financial resources and CHW interventions affect rural healthcare delivery. The case illustrates how patients modify their diets and behaviors to manage illness while revealing the household adjustments that occur during illness and the vital functions CHWs perform by delivering medical care alongside emotional support through culturally appropriate methods.

***Case 2 – Gomane***

In the rural setting of Gomane, KwaZulu-Natal, the work of community health workers (CHWs) intersects deeply with the daily realities of individuals managing multiple chronic conditions such as diabetes, hypertension, and other co-existing ailments. Within this context, the CHW plays a vital, multifaceted role that extends beyond conventional healthcare delivery. Their responsibilities include conducting household visits, monitoring medication adherence, checking child immunization status, identifying social support needs, and referring community members to institutions such as clinics, Home Affairs, SASSA, and the Department of Social Development.

The CHW’s daily routine involves meeting with patients, initiating conversations to ensure trust and confidentiality, and addressing concerns ranging from health symptoms to emotional and social stressors. They are also instrumental in navigating bureaucratic processes for undocumented individuals or those lacking essential documents like birth certificates, often assisting caregivers to obtain what is needed for child grants or social services. Their work requires diplomatic communication, particularly when addressing sensitive issues such as household hygiene, without offending the household members.

The healthcare challenges faced by individuals with comorbidities are extensive. Many struggle with lifestyle adjustments, particularly in relation to dietary changes. Foods that were once staples and sources of comfort, such as sugary drinks, cakes, or fatty meats, must be eliminated, leading to emotional and social withdrawal from family gatherings or cultural events where such foods are common. Patients describe the effort to avoid certain foods as difficult and at times, disheartening, especially when faced with constant cravings and reminders of what they can no longer consume. Despite this, some develop personal strategies, such as reducing sugar intake gradually or substituting juice for tea, though the process is rarely easy.

Medication routines are another significant burden. Patients managing multiple conditions often find the volume of pills overwhelming. This results in emotional fatigue, with some expressing feelings of helplessness or fear that their life is nearing its end. Yet, the support of CHWs is pivotal in helping them reframe their situation. Through empathetic conversations, CHWs encourage patients to accept their condition and focus on the benefits of adherence, emphasizing that their life can still be meaningful and manageable.

Trust between CHWs and community members varies. Adults are generally more open and forthcoming, while younger individuals may be more reserved, especially if they are not familiar with the CHW. Familiarity and consistent engagement are key factors in determining how comfortable a person is in disclosing sensitive information. Some patients prefer CHWs they know well, while others fear that familiarity may lead to judgment or exposure within the community. The CHW is expected to maintain confidentiality at all costs, never discussing one household’s issues with another.

Challenges within the community include logistical barriers, such as CHWs being responsible for large geographic areas with insufficient staffing. This limits the frequency and depth of engagement. Additionally, while CHWs receive some formal training, they often express the need for further education in areas such as counselling and HIV testing, as they are increasingly asked by patients to provide services outside their current scope of practice. The lack of training creates a gap in service provision, despite the willingness of CHWs to expand their role.

The absence of former partnerships with NGOs such as the Red Cross has been felt, as collaboration between healthcare and social support entities had once strengthened community outreach. Although the CHWs now operate more independently, they still engage in cooperative problem-solving when overlapping issues are identified in the field.

Within the household, the management of illness is often a collective effort. Families develop shared routines around medication schedules, diet planning, and clinic visits. Communication is generally open, and family members support each other in coping with the limitations and requirements of living with chronic illnesses. The role of household heads, especially elders, is prominent in enforcing dietary discipline and ensuring that all treatments are taken as prescribed. They face their own physical challenges but continue to provide leadership in caregiving and health management.

Despite all difficulties, community members remain resilient. Many show a strong sense of personal responsibility and commitment to living healthily, even as they acknowledge how emotionally taxing it is to continuously deny themselves foods they love or maintain complex medication regimens. They persevere not only for their own wellbeing but also for their families, navigating each day with a mix of determination, humour, and practical compromises.

The case reflects the interdependence of personal health management, social support, and community-based healthcare delivery in rural South Africa. It underscores the need for strengthened training programs for CHWs, improved staffing, and renewed collaboration with external support services. It also highlights the resilience and adaptability of both healthcare workers and patients as they strive to maintain dignity, wellness, and hope under difficult conditions.

***Case 3 – Gomane***

In the rural community of Kwanovuka in Sawongo, located in the Impendle subdistrict of KwaZulu-Natal, South Africa, community health services are delivered under challenging but resilient conditions. This deep rural area consists of widely dispersed homesteads, extensive grazing lands, and little to no industrial or formal employment infrastructure. Subsistence farming and public employment programs such as the Community Works Programme (CWP) and the Expanded Public Works Programme (EPWP) provide some level of economic activity. Infrastructure limitations, poor public transportation, and long walking distances define the geographic and logistical constraints faced by both healthcare workers and community members.

Community Health Workers (CHWs) serve as vital links between the formal health system and the local population. Their daily responsibilities include visiting households to conduct health profiling, educate community members on treatment adherence and hygiene, encourage home gardening, and refer individuals to clinics or other governmental departments. They ensure confidentiality and aim to empower families while maintaining discretion, even when discussing cases in public forums such as war rooms. Despite their commitment, CHWs work with minimal supplies. They often use their personal resources to buy exercise books or travel to clinics for gloves and other basic items. Supplies such as uniforms and proper footwear are inconsistently provided and poorly suited for the long distances they walk. In some cases, they end up serving nearly double their assigned household quotas each month, driven by a desire to ensure that no community member is left behind.

Outreach Team Leaders (OTLs), often trained nurses, oversee CHWs and handle case escalations. Their duties involve conducting home visits to verify reports, making clinical assessments, booking clinic or hospital appointments, and coordinating with various stakeholders such as SASSA, Home Affairs, and local government structures. OTLs attend war room meetings, where interdepartmental challenges are discussed and resolved. However, with only a few OTLs managing large numbers of CHWs, direct supervision is limited. This structure places significant pressure on CHWs to work autonomously while still needing OTLs for clinical interventions.

The challenges faced by CHWs are numerous and systemic. They lack dedicated communication tools after the expiration of previously provided mobile contracts, leaving them without effective channels to coordinate with supervisors or report cases. CHWs have transitioned away from providing hands-on care, such as bathing patients, toward health education due to increased community size. This shift has caused concerns in households that require more intensive care, as education alone does not always meet their needs.

Social stigma around conditions like HIV/AIDS significantly impacts service delivery. Some individuals conceal their health status or refuse to provide clinic cards out of fear that their condition will be exposed. This reluctance extends to young parents, especially those from newer generations, who may distrust health workers or feel judged. CHWs are sometimes denied access to children’s clinic cards, which impedes immunization tracking and care. These issues often require escalation through ward committees before reaching the clinic or war room level.

Despite these barriers, there is an evident passion for the work. CHWs express a deep sense of fulfillment in helping others and often go beyond their official responsibilities. They take pride in earning a living while educating and comforting families. However, they also voice frustration about their financial compensation and lack of material support. They hope for more consistent provision of supplies and recognition of their contributions.

Mobile clinics have provided some relief, especially for households that cannot afford transport to the clinic. However, these clinics visit only once a month. While CHWs collaborate with programs like CWP, which assist the elderly with cooking and laundry, there is limited interaction with other NGOs or community-based organizations. Existing collaborations, such as those with traditional healers or ward committees, typically occur during meetings or campaigns, but broader coordination remains sparse.

Efforts to improve the program could include reintroducing communication tools, ensuring regular provision of uniforms and supplies, and possibly increasing stipends. Structural integration with other community stakeholders could further strengthen the support network for both CHWs and the households they serve. The overall picture is of a committed workforce navigating complex social and logistical landscapes with limited resources but considerable compassion and resolve.

**Taylors Cases**

***Case 1 – Taylors***

The setting of this case study occurs within rural KwaZulu-Natal South Africa where the population faces difficulties accessing health care because of the limited infrastructure and harsh living conditions. The residents especially elderly people and chronically ill patients face difficulties managing their health because poor road conditions together with unreliable electricity and water scarcity limit their ability to receive essential services. Six members live in this household with the elderly woman who experienced a stroke twenty years ago becoming bedridden. The elderly woman depends completely on her caregiver who performs personal hygiene duties and gives her medication and turns her in bed and provides emotional care. The caregiver lives with the family and cares for both her chronic conditions including hypertension and diabetes. She devotes her time to care for the older woman while managing her health and preventing the development of bedsores. A senior family member actively participates in small-scale gardening activities by producing leafy greens and carrots to meet the nutritional requirements of the household. The family depends on government pension funds because there are no working members in the family. The clinic remains inaccessible to the residents of this community. The elderly woman initially moved with a wheelchair until the road damage caused wheel failure which made the chair unusable. The household must spend R100 for each car rental trip to transport the elderly woman which creates a financial strain and necessitates a minimum of four people to move her into and out of the vehicle. The household depends intensely on community health workers who perform home visits. The community health worker delivers health education while monitoring medication adherence for chronic illnesses and provides Vitamin A and deworming treatments to children and makes professional referrals when needed. The community worker teaches residents about hygiene and TB and HIV and diabetes and hypertension despite the ongoing HIV-related stigma that makes its management more complicated than other chronic illnesses. People reveal their diabetes and high blood pressure status more easily but hide their HIV status because of societal discrimination. The environment poses significant obstacles to the dedicated household members. The deteriorated state of the roads makes elderly transport both unsafe and exhausting. The household requires young members to obtain water from faraway locations since they need to bring it home. The frequent power outages during cooking require the family to consume bread while selecting less healthy alternatives. Safety remains a primary concern throughout the region because local gangs known as "amaphara" engage in theft activities and drug use. The members of this group steal household items to resell them inside their community. Residents must secure their house rooms even when moving from one area of the house to another. This family receives little to no regular support from social services which includes NGOs and social workers and public programs because they do not have any nearby. Basic facilities together with schools exist at a distant location from the community. The community has launched unstructured approaches to fight crime and youth substance abuse but the outcomes remain unclear and inadequate. The situation demonstrates the strong family bonds between members who bear heavy responsibilities and the essential yet unrecognized work of community health workers while showing how people cope with various chronic diseases in a setting without adequate infrastructure support. The household manages their challenging situation through consistent effort and dignity while providing proper care to their vulnerable members using only minimal external support.

***Case 2 – Taylors***

The case occurs within rural KwaZulu-Natal in South Africa where communities face restricted infrastructure alongside high unemployment rates and inadequate healthcare services. Community-based care relies on mobile clinics together with family caregivers and community health workers (CHWs) to deliver both preventive care and basic medical support to residents. Each household consists of multiple generations which includes elderly people who suffer from chronic diseases such as hypertension alongside diabetes and HIV/AIDS. Many elderly individuals suffer from two or more chronic illnesses while their healthcare relies on neighbors relatives or local caregivers who manage their medication and hygiene and medical clinic visits. These caregivers lack proper training and do not receive payment for their work because they face financial difficulties along with social instability. CHWs carry out their duties by checking three households daily. The community health workers educate residents about hygiene practices and disease prevention along with treatment compliance. The medical professionals verify medical cards while distributing vitamin supplements and deworming medication to children before sending unwell patients to clinics for further treatment. The work requires both physical health services and relationship-building efforts to establish trust with community members. The staff uses praise as a tool to promote healthy behaviors together with sustaining community cooperation. CHWs play an essential part in the system yet they function with limited resources. On-site blood pressure or glucose level testing becomes impossible because the CHWs do not possess medical monitoring equipment. Elderly patients who remain immobile since their stroke experiences major obstacles to reach clinics because of their immobility. CHWs depend on family members to transport patients since they do not possess mobile diagnostic tools. These communities receive their health services through both permanent clinics and mobile healthcare units. The residents receive transportation to clinic facilities or mobile health stations through the assistance of their neighbors and relatives whenever they can move. Some patients need scheduled injections as part of their medical treatment. Medical routines become disrupted along with patient health risks when caregivers leave their posts or become unavailable. An elderly male patient received his medication injections through his granddaughter until she vanished unexpectedly. A medical process became clear to the daughter-in-law after she received instructions through phone calls to perform the treatment which showed how easily informal caregiving arrangements fail. The existing social problems make the situation worse. The high unemployment levels combined with widespread substance abuse among young males have created a rise in criminal activities throughout the area. The local population uses alcohol and woonga as well as other drugs while substance-dependent individuals steal property and commit violent crimes to sustain their drug habits. The crime rate increases most during times with power outages as well as the early mornings and late evenings. Theft of flat-screen TVs represents one of the many burglaries that occur since perpetrators focus on stealing contemporary items. The residents of these areas have implemented burglar guards while maintaining complete home locking as a security measure. The residents sometimes take matters into their own hands because they do not trust police to solve their problems effectively. Resident communities show active survival capabilities despite the existing obstacles. Households maintain vegetable gardens to provide food security regardless of their financial situation. Older adults participate in subsistence farming activities which helps support household nutrition needs. Community water access, however, remains inconsistent. The communities built their own water supply systems through springs and boreholes and shared water tanks because formal municipal water delivery stopped in some regions. The care system functions through an unsteady network of unpaid labor combined with community support and limited state resources. The community health workers maintain this system but face limitations because they lack essential resources and equipment. The rural care for people with multiple chronic conditions requires enhanced mobile diagnostic tools as well as training for informal caregivers and comprehensive social programs to fight crime and substance abuse.

***Case 3 – Taylors* *(There is no OTL but CHWs are supervised by the OM)***

KwaNdeleshane residents in rural KwaZulu-Natal receive vital healthcare through community-based healthcare because they face both chronic illnesses and extensive poverty in the area under Taylors Clinic. The system depends heavily on Community health workers (CHWs). The daily duties of the workers include educating residents through home visits and monitoring medication adherence as well as promoting hygiene practices and establishing self-sufficient home gardens. They oversee child-headed households and track immunization compliance while detecting signs of neglect or abuse which they then report to social workers. The workers handle all aspects of care including healthcare responsibilities as well as informal social work activities and community dispute resolution services for cases involving elderly abuse and coercive relationships. The essential work of CHWs remains hindered by multiple severe restrictions. The workers need basic supplies including gloves nappies stationery and bandages but they rarely receive these necessities. The clinic faces difficulties because screening devices for chronic diseases including hypertension and diabetes are insufficient and must be distributed between all staff members. The absence of transportation to coordination meetings such as the “war room” prevents staff from participating in vital community planning sessions. The provided uniform remains insufficient and inappropriate for weather conditions and the heavy shoes make walking long distances difficult. The CHWs must spend their own money to buy stationery items and pens and notebooks. When food parcels and nappies are distributed through CHWs it sometimes produces negative reactions from the community and puts CHWs at risk. The CHWs face accusations from residents who believe they are withholding support and favoring particular households which leads to conflict and distrust. The situation has caused CHWs to stop working on selected projects which forced them to pass their responsibilities to councillors or traditional leaders. Caregivers must handle their individual difficulties at home. An unemployed caregiver devotes herself completely to her elderly relative who suffers from multiple chronic illnesses as she spends her days preparing food and caring for hygiene and giving medicine to the patient. She uses gloves and adult nappies for managing symptoms of diarrhoea while purchasing these items herself after health workers run out of supply. She takes full responsibility for the elder's welfare by handling all household duties and medical trips to the clinic through family car transportation. She did not encounter personal difficulties reaching the clinic but she observed the extensive pressure of caring without assistance from outside services particularly when public health items such as gloves and nappies remain unavailable. The management team at Taylors Clinic encounters systematic restrictions during clinic operations. The healthcare facility faces two major problems: employees leave for superior positions and the medical staff remains insufficient. The facility faces two major problems: insufficient space for male consultations and general overcrowding. The clinic controls skill deficiencies through periodic training events although it requires more organized structured development programs. The data management systems are based on paper files which causes delays because of power interruptions and poor networking systems. Temporary medicine shortages are addressed by establishing connections with neighboring healthcare facilities. The clinic treats mostly chronic diseases such as HIV and TB together with diabetes and hypertension. The clinic patients who have multiple conditions tend to stop their treatment because they cannot afford food or transportation to reach the clinic. Elderly patients along with those who are severely ill fail to attend their scheduled appointments because they lack proper transportation and support. The clinic sends patients to social workers but home visits are difficult because CHWs lack access to transport and phones. Each chronic illness has separate treatment guidelines yet staff members at the clinic strongly endorse integrated care that combines treatment protocols with appointment scheduling. The implementation of this approach would decrease patient burden and enhance treatment adherence. The community health workers together with nurses perform home visits to evaluate residential environments and create specific recommendations. The CHWs in the community perform screenings which direct high-risk people to either clinic appointments or home outreach programs. Health literacy development along with youth support groups receives attention but the program lacks organized care for patients who have multiple medical conditions. Rural healthcare functions as the fundamental support system in rural areas yet it lacks structural backing despite its critical role in maintaining operations. The lack of recognition together with communication tools deficiency and transportation problems creates high burnout risks for CHWs and caregivers. Community health workers along with informal caregivers sustain the current system through their dedicated efforts although systemic investment remains an urgent requirement. The system requires proper investments for transport systems and resource management and digital documentation and official caregiver support programs and caregiver recognition. The development of a people-focused community health system requires an integrated approach to create resilience for rural South Africa's residents with chronic conditions.
