# Supplementary material for: A realist perspective on optimizing community health workers’ roles and functions to deliver integrated people-centred care
Source: PLOS Glob Public Health. 2025 Sep 3;5(9):e0004926. doi: 10.1371/journal.pgph.0004926 (PMC12407478; doi:10.1371/journal.pgph.0004926)
Supplement: S2 File — (DOCX) [file pgph.0004926.s002.docx]

**AI Prompt**

**Prompt code to identify emerging CMOs**

*“What are the* ***Context*** *(as Pre-existing structures, settings, environments, circumstances, or conditions that shape whether certain behavioural and emotional responses,* ***Mechanisms*** *(as An element of the actor's reasoning towards an intervention such as beliefs values desires feelings perceptions causal force and cognitive processes - mechanisms can't be an actual act, they are how people respond or reason to a particular intervention or context) and* ***Outcomes*** *(as the observable change or result that occurs due to an intervention, considering both intended and unintended effects, and taking into account the context and mechanisms that produced those changes) for each reference - include multiple quotes where they are and summarize patterns using context, mechanism, and outcome”*

**AI-Supported Reasoning and Example Prompts**

**1. Example Prompt to DeepSeek (R1 Reason Feature):**

*“Given this transcript excerpt, what mechanism may be operating in response to this context: “If you enter this household with a community health worker from this area, that is the only time you will get responses.”*

**AI-Generated Response using R1 feature:**

*This quote suggests that CHW embeddedness—being locally known or culturally familiar—is a critical contextual condition for household engagement. The presence of a familiar CHW appears to reduce perceived risk and create a sense of safety or legitimacy, enabling trust and openness. The mechanism at play may involve recognition, cultural affinity, or credibility attributed to local actors.*

**2. Researcher Validation Note:**

*This interpretation supported emerging themes around “familiarity,” and “embedded trust,”. The research team validated this mechanism by comparing it to other cases across household interviews and observations where local CHWs facilitated entry or acceptance, while outsiders were met with reluctance or silence. This was further validated using existing literature to see whether this was ever observed elsewhere and the context it occurred under.*

**3. Role in CMO Configuration:**

**Context:** CHW is locally known in the community

**Mechanism:** Familiarity and embedded trust reduce defensiveness

**Outcome:** Household members feel safe to respond and engage openly with services
