# Supplementary material for: A realist perspective on optimizing community health workers’ roles and functions to deliver integrated people-centred care
Source: PLOS Glob Public Health. 2025 Sep 3;5(9):e0004926. doi: 10.1371/journal.pgph.0004926 (PMC12407478; doi:10.1371/journal.pgph.0004926)
Supplement: Attachment — Submitted filename: Reviewer report.docx [file pgph.0004926.s005.docx]

Title: The title reflects the focus of the article.

Abstract: Is reflective of the content of the article.

**Introduction**
The introduction section is well-structured and concise while providing sufficient information to offer a comprehensive overview.

The authors use numerous abbreviations, which may become confusing for the reader, as they need to repeatedly refer back to their definitions. Consider reducing the use of abbreviations that appear infrequently by writing them out in full, such as OTL and OM.

The problem is clearly articulated.

**Methodology**
The researchers should be commended for employing a realistic evaluation approach, as it provides a novel perspective on a phenomenon that has already been explored in the literature.

Overall, the methodology is reported in detail; however, some sections lack clarity. For example, in Step 1, the practical application is not clearly outlined. In Step 2, it is unclear what information was gathered during each data-gathering activity. Given the multiple data collection methods (interviews, observations, and focus groups), it is difficult to determine which data was collected at each stage. Additionally, the process of recording and analysing observations is not clearly explained.

The data-capturing methods and opportunities are extensive and complex. Consider summarising this information in a diagramme or table that provides a visual overview of the data sources, data collection tools/methods, and the focus of each data-gathering activity (i.e., what each interview, observation, or focus group sought to collect in response to the research question). This could enhance the accessibility of the methodology for a broader audience.

It is also unclear whether the coding approach was inductive or deductive. See specific comments related to other steps.

It is also unclear whether the coding approach was inductive or deductive. See specific comments related to other steps.

**Results**
Why was no demographic information included?

Overall, the results are comprehensive and detailed. However, the volume of information presented may be overwhelming, making it difficult to track all findings. It might be beneficial to divide the results into two separate articles. Please refer to the manuscript for specific comments on this section.

**Discussion**
The discussion is comprehensive and includes appropriate detail.

**References**
Please update all references to correctly reflect the use of "et al."

**Future Research**

It would also have been valuable to compare governance, access to resources, and other factors across the five different communities to examine their influence on role performance.
