# Supplementary material for: A realist perspective on optimizing community health workers’ roles and functions to deliver integrated people-centred care
Source: PLOS Glob Public Health. 2025 Sep 3;5(9):e0004926. doi: 10.1371/journal.pgph.0004926 (PMC12407478; doi:10.1371/journal.pgph.0004926)
Supplement: Attachment — Submitted filename: Response to Reviewers.docx [file pgph.0004926.s007.docx]

**Response to Reviewers – Manuscript ID PGPH-D-25-01197**

**Dear Editor and Reviewers,**

We thank you for your careful review of our manuscript titled *“A Realist Perspective on Optimizing Community Health Workers’ roles and functions to deliver Integrated people-centred care.”* We are grateful for the insightful and constructive comments provided. We have revised the manuscript accordingly and addressed each comment in detail below. Our revisions begin with a response to the editor’s comments (section 1), followed by a set of responses addressing in-text comments from the manuscript, including corresponding line numbers and explanations (section 2). Finally, it concludes with detailed, point-by-point responses to the individual reviewers’ comments (section 3).

Changes made in the manuscript are highlighted using track changes. A clean version was also submitted as required.

**1. Response to the Editor comments**

| # | Editor Comment | Action Required / Proposed Response |
| --- | --- | --- |
| 1 | Data Availability Statement requires revision to comply with PLOS policy. Current version lacks a non-author contact, minimal data set access, and lists an embargoed Figshare file. | The data availability statement has been revised. There is a direct link where the data can be accessed, and the embargo has been lifted. A non-author contact has also been provided. |
| 2 | Financial Disclosure requires full sentences and initials per funder. Also state funder role. | The Financial Disclosure Statement has been updated to include author initials, and a statement on the funder's role has been added. |
| 3 | Funding sources and grant numbers must match across the Financial Disclosure and Funding Information sections. | Funding sources and grant numbers have been cross-checked and revised to ensure that they match. |
| 4 | Figures must be submitted as separate .tif or .eps files under 10MB, not embedded in manuscript. | Figures have been converted and submitted separately as .tif as per request |
| 5 | Figure 3 may include copyrighted images. Confirm if images were hand-drawn or provide license/permission. | The figure has been redrawn by the authors, who therefore retain the copyright. Additionally, the content is original and authored entirely by the study team. |
| 6 | Please include a complete copy of PLOS’ questionnaire on inclusivity in global research in your revised manuscript. | We have completed the PLOS questionnaire on inclusivity in global research and uploaded it under supporting Information with the revised submission. |
| 7 | “Unpublished data" is not permitted under PLOS' data access policy. | The unpublished reference that was cited has now been published and is therefore referenced as a published article in this revised submission. |

**2. Revisions addressing reviewer comments and suggestions that appear in the manuscript.**

| Line No. | Reviewer Comment | Author Response | Action Taken |
| --- | --- | --- | --- |
| 82 | Consider changing to "data capturing." | Thank you. We have revised the phrase to "data capturing" for clarity. | Revised |
| 84 | It is unclear what is meant here? Is the idea really to integrate CHC into formal health facilities? Or into formal health care provision/ services? | Thank you for this helpful clarification request. The intention is to strengthen linkages and coordination between community-based care and formal health services, not to integrate into health facilities themselves. We have revised the sentence for clarity. | Revised |
| 91 | Please add "the" | The missing "the" has been inserted as suggested. | Revised |
| 94 | For what purpose? | The purpose is stated at the end of the sentence - “to achieve accessible, responsive and people-centred care”. | Not Revised |
| 135 and 136 | Why only look at the interaction between CHWs and household if you want to evaluate the effectiveness of the programme. There are also contextual barriers, social and institutional barriers that negatively impact on achieving outcomes. | We agree with the reviewer that evaluating programme effectiveness requires a comprehensive understanding of not only interpersonal interactions but also the broader contextual, social, and institutional factors.  The study used CHW–household interactions as its main data collection entry point. However, data collection included more than just household interviews but went on to include: structured observations of CHW visits, interviews with Outreach Team Leaders (OTLs), Clinic Operational Managers, focus group discussions with CHWs and household decision-makers. This allowed us to explore broader contextual, social, and institutional factors.  The authors have added this clarification to the revised manuscript in line 138 and 139. It also appears in section 2.3.1 “(Sampling and participant recruitment)”. | Revised |
| 151 | From a methodological perspective, it is not clear how step 1 was done practically. How and when was this done and by whom? | Thank you for seeking clarity. We have referenced a published manuscript by the first author which describes this section in detail as part of the same body of work. Full details of the process can be found there, should readers wish to get a step-by-step process. This has now been added in this manuscript as well. | Revised |
| 203 | How were communities selected? Convenience? | We clarified that selection was based on clinic catchment areas and ongoing CHW activity, with input from operational managers through purposive sampling technique. This already exists in the manuscript – therefore no revisions were made | Not revised |
| 208 | Please explain the application of these to the reader. | Expanded the explanation of the NICE guidelines and their alignment with people-centred care. | Revised |
| 204 | Why only 15 households? | We found that after 15 cases, data reached saturation in terms of identifying new CMO patterns. This is consistent with realist evaluation standards, where sampling is guided by explanatory depth and theoretical saturation - not statistical representativeness. (There was no need for revision in the manuscript, however, clarity was required by the reviewer) | Not Revised |
| 209 | how did you prevent selection biaise here? CHWs selecting specific households that might have successful outcomes, etc. | We were not evaluating CHW performance on specific health outcomes, but rather we were exploring the underlying mechanisms through a realist inquiry, therefore, CHWs did not know which mechanisms were being studied or whether we were looking for any pre-determined context that they might have influence over certain mechanisms to achieve specific outcomes | Not revised |
| 215 | How was the leader identified? If that person was working, what happened then? Who did the interviews for all participants? | We appreciate the reviewer’s question. A relevant paragraph has been added to the manuscript to give more details. The study team did the interviews, and we listed them in the manuscript. | Revised |
| 221 | What was the aim of these interviews and focus of the questions? | Added a paragraph that explains aim of these interviews and focus of the questions. | Revised |
| 246 | why only 3 of the 5? Convenience? | FGDs were conducted in three of the five sites based on the principle of theoretical saturation. By the third FGD, no new context-mechanism-outcome (CMO) configurations were emerging, and the data were consistently reinforcing previously identified themes. This saturation point indicated that further FGDs were unlikely to yield new explanatory insights. Therefore, the decision to stop at three FGDs was methodological and aligned with realist evaluation and not based on convenience. | Not Revised |
| 261 | Please reference your methodology in this section | Included relevant citations and references for the data analysis methods. | Revised |
| 274 | For readers not familiar with technology, please explain in more detail | Added further explanation about the use of AI tools (DeepSeek) for coding and reasoning. | Revised |
| 272 | what is meant with "model" here? Do you mean programme/ tool/ application? | Changed “model” to “AI tool” for clarity. | Revised |
| 322 | Explain abbreviations | Abbreviations of cases have been explained in appendix 1 “case descriptions”. | Revised |
| 331 | Are CHWs considered nagging or gossips? Clarify | Clarity provided: CHWs were considered as “nagging” in BUE-HDM-Case 1 and as gossiping in GME-FGD therefore both. | Revised |
| 334 | what is unclear is the origins of the beliefs/ perceptions that they are not trustworthy because they are volunteers or gossips. Where these explored in interviews and focus group | These were not further explored but one would argue that this was a combination of both as later on in the manuscript it is shown that trust is not only relational but can be influenced also by professional legitimacy. | Revised |
| 354 | Same person said both? | Thank you for picking this up. This has been corrected; indeed, it was the same person that said both. | Revised |
| 356 | Quotes don’t support conclusion | Agreed. However, we have removed this section because the point it made was already covered earlier, in a more appropriate context with a quote that directly supported the conclusion being drawn. | Revised |
| 403 | Please put this section in context for the reader. Is food for 5 people too little? or do you mean that 5 food parcels were given, thus too few for the need in the community | This has been clarified *“too few for the need in the community”* | Revised |
| 404 | Should this be the role of CHWs? To provide the resources the community need? Is that expectation not setting CHWs up to fail? Thus is more resources and better supply chains for CHWs really the answer? The best solution? | We thank the reviewer for this critical insight. We agree that expecting CHWs to directly provide material resources can risk setting unrealistic expectations which may contribute to role overload and reinforce systemic neglect. In response, we have revised the section to reflect this concern. Rather than promoting improved supply chains as the sole solution, we now emphasize the importance of clarifying CHW roles, strengthening referral pathways to social services, and ensuring institutional support (e.g. supplies, materials, etc.) so that CHWs are not the de facto providers of material support but the community understands that it’s the job of institutions. | Revised |
| 406 | Explain the figure in more detail | The figure is meant to visually show the dynamics that are being narratively explained and supported by quotes – we believe frequently referencing the diagram together with the quotes will confuse the reader | Not Revised |
| 415 | It is unclear what evidence the authors are referring too here. Please clarify for the reader. The information under section 3.1? If so, which parts? | This has been clarified that the evidence is from section 3.1. and its overarching and informs the middle-range theory generated in section 3.2. | Revised |
| 514 | Engagement in what? Data collection? | This has been clarified to explain engagement with the health system and communities | Revised |
| 587 | These sounds like curative outpatient services? Please make it explicit show it contributes to health promotion | Clarified to preventative and health-promotion aspects of community – and home-based care duties. | Revised |
| 534 | how and who identified the outcomes. This was not reported on above | Thank you for this valuable observation. A paragraph has been added in the methods (section 2.4) to explain how and who identified the outcomes. | Revised |

**3. Response to individual Reviewers**

**Reviewer #1**

We appreciate Reviewer 1’s constructive suggestions on improving the presentation and alignment of the findings and discussion. These helped us improve the coherence and impact of the manuscript.

| Reviewer Comment | Author Response | Location in Manuscript |
| --- | --- | --- |
| The presentation on findings could be made in more detail. Likewise, discussions could be aligned with the findings and with current advancements in the area. Besides that the manuscript is presented well. | We clarified key findings and enhanced alignment between the discussion and added more references and current advancements. | Results and Discussion Sections |

**Reviewer #2**

We thank Reviewer 2 for their helpful and specific recommendations on improving the abstract, discussion, and figure presentation. We have addressed each suggestion as follows:

| Reviewer Comment | Author Response | Location in Manuscript |
| --- | --- | --- |
| Abstract: Mentioning of qualitative thematic coding and or qualitative CMO synthesis could strengthen the clarity for; clarify in the abstract that it is a qualitative study. | Revised the abstract to clearly state that the study is qualitative, utilized thematic coding and CMO synthesis. | Abstract |
| Discussion: Consider adding a paragraph at the end to clearly summarise study constraints such as sampling scope, generalisability, and limitation of analysis techniques, etc. | We have added a paragraph summarising limitations regarding sample size, generalizability, and analytical approach. | Discussion, final paragraph |
| In the final submission, ensure figures (Fig 1–7) are fully integrated and properly captioned; they are crucial to illustrating CMO configurations and program theories. | We reviewed all figures, ensured integration and improved captions to better explain the visualized content. | Figures 1–7 |

**Reviewer #3**

**We thank Reviewer 3 for their detailed feedback, particularly regarding data availability, AI methods, and policy relevance.**

| Reviewer Comment | Author Response | Location in Manuscript |
| --- | --- | --- |
| Data Availability: Update the Data Availability Statement to reflect that the data will be fully and openly accessible on Figshare upon publication, providing a direct link or accession number. Clarify that no separate request to the supervisory team will be required for access if the data is indeed made publicly available. | We have updated the Data Availability section to confirm public access via Figshare upon publication, with a direct link provided. | Data Availability Statement |
| AI Methodology Details: Consider adding a supplementary appendix or expanding the methods section to include specific examples of the prompts used for DeepSeek AI and a more detailed explanation of how the "R1 reason feature" was employed and its outputs interpreted and validated. | We have expanded the methods section and included a supplementary appendix with prompt examples and a detailed explanation of the R1 feature and its interpretation process. | Methods and Supplementary Material |
| Policymaker Perspective: While acknowledged as a limitation, the discussion and conclusion could further emphasize how the identified governance challenges and proposed middle-range theories directly inform and guide policy reforms, even without direct input from high-level policymakers in this particular study. | We have added a paragraph on “policy implications” to inform and guide policy reforms | Discussion |

**Reviewer #4**

We appreciate Reviewer 4’s positive remarks and helpful suggestions. In response to reviewer feedback, we revised the manuscript to improve clarity, reduce redundancy, and strengthen alignment with existing literature. We also expanded the discussion to include actionable policy implications and added a section on potential AI bias in data analysis.

| Reviewer Comment | Author Response | Location in Manuscript |
| --- | --- | --- |
| The manuscript is largely easy to read and generally well-written in standard English. However, there are areas where conciseness could be improved. For instance, in the discussion section, revisiting previously explained mechanisms without adding new interpretation may lengthen the manuscript unnecessarily. Avoid unnecessary repetition to reduce redundancy. | We thank the reviewer for this thoughtful feedback. We carefully reviewed the discussion section and revised several paragraphs to improve clarity and conciseness. In particular, we removed redundant summaries of previously discussed mechanisms unless they were linked to new interpretation, synthesis across MRTs, or implications for policy and theory. These changes help tighten the narrative while preserving analytical depth. | Discussion |
| It requires more discussion on how the findings align or contradict existing literature. | We have expanded the discussion with more explicit comparisons between our findings and recent literature to highlight alignment and divergence. | Discussion |
| Consider offering more concrete policy implications that stakeholders could implement based on your findings. | Added a subsection in the discussion detailing actionable policy implications | Discussion |
| Briefly discuss how the use of AI in data analysis may influence bias. | We have added a paragraph discussing the potential for AI bias and described measures we took to mitigate this risk. | Discussion Limitation Section |
| The presentation and labeling of the tables could be improved. | All tables have been revised for clarity, consistent labeling, and improved readability. | Tables |

**Reviewer #5**

We thank Reviewer 5 for their encouraging review and support of our manuscript.

| Reviewer Comment | Author Response | Location in Manuscript |
| --- | --- | --- |
| The manuscript is concise and details its findings in a presentable manner. It presents evidence useful in the relevant area and is recommended for publication in the journal at present. Ethical concerns have been met. | Thank you for your positive feedback. We are pleased the manuscript meets expectations. | — |

**Reviewer #6**

We appreciate Reviewer 6’s support and recognition of the study’s theoretical and policy contributions.

| Reviewer Comment | Author Response | Location in Manuscript |
| --- | --- | --- |
| The authors offer thoughtful and policy-relevant findings drawn from a realist evaluation... | Thank you for your encouraging feedback. No revisions were necessary or required. | — |

**Reviewer #7**

We addressed reviewer suggestions by reducing unnecessary abbreviations and enhancing methodological transparency. Clarifications were added regarding data collection steps, and a summary table was added for clarity. We also improved readability of the results through restructuring and ensured proper reference formatting throughout.

| Reviewer Comment | Author Response | Location in Manuscript |
| --- | --- | --- |
| The authors use numerous abbreviations, which may become confusing... Consider reducing the use of abbreviations... | We reviewed the manuscript and removed or expanded rarely used abbreviations to enhance clarity. | Throughout Manuscript |
| In Step 1, the practical application is not clearly outlined. In Step 2, it is unclear what information was gathered... | We have provided a reference that provides full details of step one that comes from the same body of work. We have provided an additional data collection table to provide more clarity for the reader. | Methods Section |
| Consider summarising data collection in a diagram or table. | A summary table of data sources, methods, and focus areas has been added. | Methods Section and Table 2 |
| Clarify whether the coding approach was inductive or deductive. | Clarified that coding followed an inductive approach with iterative refinement and retroduction. | Methods Section |
| Why was no demographic information included? | This data was not collected, and we have added that to the limitations section as this would have provided more nuanced understanding of the results. | Results Section |
| The results are comprehensive but may be overwhelming. Suggest dividing into two articles. | Thank you for this suggestion. However, we have maintained a single article format but tried by all means structure the results into clear subsections for enhanced readability. | Results Section |
| Update references to reflect use of "et al." | All references have been reviewed and corrected for formatting. | References Section |
